# How Adaptation Makes Low Firing Rates Robust

**DOI:** 10.1186/s13408-017-0047-3

**Published:** 2017-06-24

**Authors:** Arthur S. Sherman, Joon Ha

**Affiliations:** Laboratory of Biological Modeling, National Institutes of Health, 12A South Drive, Bethesda, MD 20892 USA

**Keywords:** Adaptation, SNIC bifurcation, Firing rate

## Abstract

Low frequency firing is modeled by Type 1 neurons with a SNIC, but, because of the vertical slope of the square-root-like *f*–*I* curve, low *f* only occurs over a narrow range of *I*. When an adaptive current is added, however, the *f*–*I* curve is linearized, and low *f* occurs robustly over a large *I* range. Ermentrout (Neural Comput. 10(7):1721-1729, 1998) showed that this feature of adaptation paradoxically arises from the SNIC that is responsible for the vertical slope. We show, using a simplified Hindmarsh–Rose neuron with negative feedback acting directly on the adaptation current, that whereas a SNIC contributes to linearization, in practice linearization over a large interval may require strong adaptation strength. We also find that a type 2 neuron with threshold generated by a Hopf bifurcation can also show linearization if adaptation strength is strong. Thus, a SNIC is not necessary. More fundamental than a SNIC is stretching the steep region near threshold, which stems from sufficiently strong adaptation, though a SNIC contributes if present. In a more realistic conductance-based model, Morris–Lecar, with negative feedback acting on the adaptation conductance, an additional assumption that the driving force of the adaptation current is independent of *I* is needed. If this holds, strong adaptive conductance is both necessary and sufficient for linearization of *f*–*I* curves of type 2 *f*–*I* curves.

## Introduction

One of the striking features of neuronal spiking is that many neurons fire at low rates near threshold and robustly resist increasing their firing rates when driven *in vitro* by an applied current. In early observations Hodgkin noted that there was a sub-population of neurons that could fire at arbitrarily low rates near threshold [1]. He called these Class I neurons to distinguish them from Class II neurons, which have a minimum firing frequency well above 0. With properly chosen parameters, the Hodgkin-Huxley equations can exhibit both classes of behavior, and theoretical analysis has identified these two cases with two distinct bifurcations leading to periodic solutions, saddle node on an invariant circle (SNIC) and Hopf bifurcation (HB), respectively [2, 3]. This is an elegant classification scheme, but is of limited help in accounting for the robustness of low-frequency firing; the firing rate is only low very near the SNIC bifurcation because

$$f(I) \propto\sqrt{(I - I_{0})}, $$

where $I - I_{0}$ is the distance from the threshold applied current [2]. In particular, the derivative of the *f*–*I* curve is infinite at the bifurcation, so large changes in frequency are seen with small increments of current.

An alternative approach is to focus on adaptation currents that provide negative feedback to slow the firing rate. This respects the physiology of slow-firing neurons and is also needed to have actual adaption—the slowing of firing rate over time during a maintained stimulus. However, as shown in detail in [4], the presence of particular currents (they considered the A-type $\mathrm{K}^{+}$ current) proposed by [5] is neither necessary nor sufficient to have low-frequency firing. They again emphasized the role of SNIC bifurcations.

The two approaches were married by theoretical analysis of HH-type models with various adaptation currents appended [6–9]. Our starting point is [9], where it was argued that the infinite slope of the *f*–*I* curve at the SNIC is, paradoxically, responsible for the ability of the adaptation current to reduce the firing rate and, moreover, accounts generically for the linear *f*–*I* curve of the adapted system.

We take another look here at spiking systems that have SNICs and are augmented with a slow adaptation variable, using phase-plane and bifurcation analysis. We aim in part to answer the question of why the lack of robustness is not merely transferred to the parameters of the adaptation variable and identify a geometric condition for avoiding this. We find that a SNIC in the system without adaptation does promote linearization, but that the *f*–*I* curve in the presence of adaptation may be linear only over a small interval unless the conductance of the adaptation current is sufficiently large. Finally we find that a SNIC is not necessary; type 2 systems in which oscillations arise from a Hopf bifurcation [2] can also show robust adaptation and linearization, though not generally low-frequency firing, if certain conditions hold.

## Results

We consider first a very simple model with polynomial expressions instead of ionic currents, Hindmarsh–Rose (HR) [10], that has the essential components, a fast spiking subsystem and a slow adaptation variable. The adaptation equation is linear, which simplifies the application of averaging. We then extend the approach to a conductance-based HH-type model, Morris–Lecar (ML) [11], by reducing it to a form very similar to that of HR. This will show that linear adaptation, while convenient for the analysis, is not required for the effect.

### Slow Firing in the Hindmarsh–Rose Model

The slightly modified HR system we use is

1 $$\begin{aligned} {dx \over dt} =& y - a(x - \theta)^{3} + b(x - \theta)^{2} + I - z \equiv F(x, y)+ I - z, \end{aligned}$$

2 $$\begin{aligned} {dy \over dt} =& \phi\bigl(c - dx^{2} - y \bigr) \equiv\phi \bigl(g(x) - y\bigr), \end{aligned}$$

3 $$\begin{aligned} {dz \over dt} =& \epsilon\bigl(s(x - \bar{x}) -z\bigr), \end{aligned}$$

where *x* represents a non-dimensional membrane potential, *y* is a fast recovery variable, like *n* in HH or ML, and *z* is a slow negative feedback variable. HR was adapted from the Fitzhugh–Nagumo model (FHN) to make the oscillations look more neuronal, with brief spikes and a long interspike interval, in contrast to the square-wave oscillations of FHN, which look more like cardiac action potentials. This was achieved by making the *y* equation quadratic rather than linear. The slow variable *z* is responsible for adaptation; it has also been widely used to study bursting, but that will not be considered here.

Equations (1), (2) constitute the fast, non-adaptive spiking system (or the “unadapted system”). Parameters are listed in Table 1. Our main interest will be to study how the firing rate depends on the applied current *I*, as modified by the adaptation variable *z*. The *z* equation (3) is slow ($\epsilon=0.0005$), and the adaptive current parameter *s* will be varied to study its effect on the *f*–*I* curves. *x̄* is adjusted to locate the threshold for spiking at $I=0$.

**Table 1:** **Parameter values for SNIC and Hopf bifurcations with Hindmarsh–Rose model**

	SNIC	Hopf
*I*	[0,20]	[−0.8,22]
*a*	1	1
*b*	3.5	3.5
*c*	1	1
*d*	5.5	5.5
*ϵ*	0.0005	0.00005
*ϕ*	0.1	0.1
*x̄*	−1.11	−0.63
*θ*	0	0.13

**Fig. 1:**
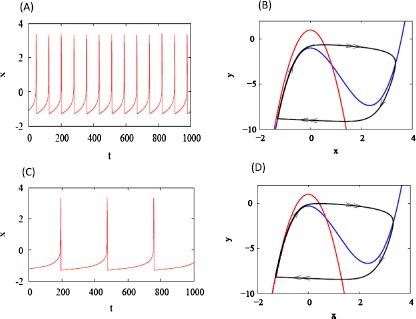
Solutions of Eqs. (1), (2). $I = 1$ and *z* fixed at 0 (**A** and **B**) or 0.7 (**C** and **D**). Time courses shown in (**A** and **C**). In the phase planes (**B** and **C**) the *blue curves are* the *x* nullclines, *red curves* are *y* nullclines, *black curves* are the trajectories, labeled with slow (*single arrow*) and fast (*double arrow*) segments. Increasing *z* narrows the gap between the left branches of the *x* and *y* nullclines, increasing the period by prolonging the interspike interval

To illustrate the effect of adaptive currents on the firing rate curve in the *x*–*y* phase plane, we first freeze *z*. When $z = 0$ (Fig. 1A) spiking is much faster than when $z = 0.7$ (Fig. 1C). The phase planes (Fig. 1B, C) show why: the gap between the left branch of the *x*-nullcline and the *y*-nullcline shrinks when *z* is increased.

**Fig. 2:**
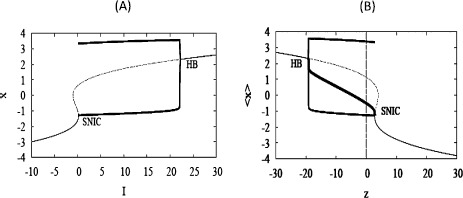
Bifurcation diagrams for Eqs. (1), (2). Bifurcation parameter *I* (**A**) or *z* (**B**). (**A**) The *S*-*shaped curve* (*grey*) shows the steady states, stable (*solid*), unstable (*dashed*). The periodic branch (*black, thick*) emerges from a Hopf bifurcation (HB) and terminates at a SNIC. (**B**) The diagram with respect to *z* is a reflection of the one for *I*. In addition, the *thick solid line* connecting HB and SNIC shows the value of *x* averaged over a spike, $\langle x \rangle$

Hindmarsh and Rose described this as the “narrow channel” mechanism because the trajectory is slow when it moves through the region between the nullclines. The current view is that the narrow channel is the ghost of the SNIC created when the nullclines become tangent at a slightly larger value of *z* or, equivalently, for a smaller value of *I* (Fig. 2).

### Approximating the Adapted Firing Rate Geometrically

**Fig. 3:**
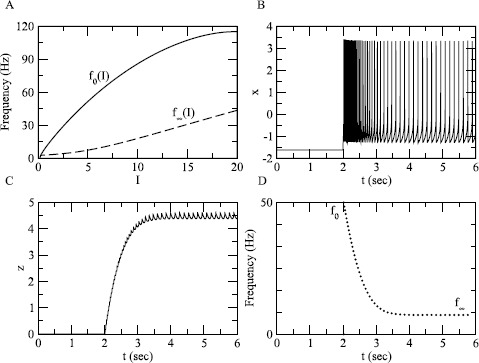
Adaptation and firing rate curves. (**A**) The unadapted *f*–*I* curve ($f_{0}(I)$; *solid*) and the steady-state adapted firing rate curve with $s = 22$ ($f_{\infty}(I)$; *dashed*). (**B**)–(**D**): Adaptation in response to a step of *I* from 0 to 5 after equilibration with adaptation turned off. (**B**) *x*; (**C**) *z*; (**D**) frequency obtained as reciprocal of interspike interval

In order to study adaptation, we now let *z* be a slow variable, and carry out a geometric fast-slow decomposition of Eqs. (1)–(3). With *z* dynamic, the system shows adaptation as *z* increases in response to a step of *I* (Fig. 3B). Fig. 3A shows the *f*–*I* curve of the fast subsystem without adaptation ($f_{0}$, solid) and the steady-state *f*–*I* curve when adaptation is turned on ($f_{\infty}$, dashed). The steady-state curve consists of the frequencies approached by the solution as $t \rightarrow\infty$ at each value of *I*. Figure 3A shows the curves as a function of *I* extracted from the bifurcation diagram of either the fast subsystem without adaptation or of the full system, and Figs. 3B–D show the approach to the steady state for $I = 5$; the empirical frequency (reciprocal of the interspike interval) in panel D agrees with the values predicted in panel A.

**Fig. 4:**
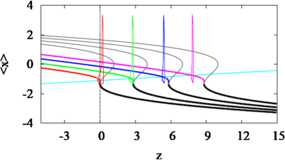
Bifurcation diagram with respect to *z* shifts with *I*. The curve of average *x*, $\langle x \rangle$, starts at HB to the left of the frame and terminates at SNIC. Colors correspond to $I=0.18, 3, 6, 9$, left to right. The trajectories evolve closely around the intersection points of the average curves and the *z*-nullcline (*cyan*). $\bar{x} = -1.1$ and $s=22$ for all *I*

Figure 4 shows how the bifurcation diagram of *x* with respect to *z* shifts to the right as *I* increases. This is evident algebraically, as the equation of the *z*-shaped curve is $z=F(x,y) + I = F(x,g(x)) + I $, where *x* and *y* are set to steady state. Then ${\partial z \over \partial I} = 1$, so the bifurcation diagram shifts to the right in the *z*–*x* phase plane.

We view this as a pseudo-phase plane for the full three-variable system and superimpose the steady-state (adapted) spiking solutions, which are accurately predicted by the intersection of the curve of average *x*, $\langle x \rangle$ in Fig. 4.

**Fig. 5:**
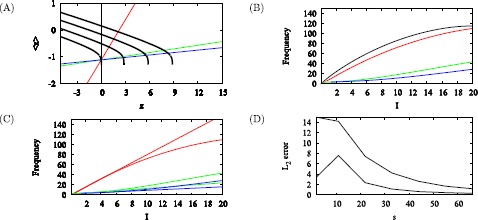
Effect of *z* nullcline slope. (**A**) Decreasing the slope shifts the intersections with the $\langle x \rangle$ curve. This results in larger steady-state *z* and lower firing rate. Nullclines drawn for $s = 2$ (*red*), $s = 22$ (*green*), and $s = 33$ (*blue*); $\langle x \rangle$ drawn for *I*= 0.18, 3, 6, 9, increasing to the right. (**B**) $f(I)$ for the system without adaptation and the three slopes in (**A**). (**C**) Least squares linear fits (*thin*) for the three *f*–*I* curves (*thick*) in (**A**) over intervals of length 6 starting at the threshold for the corresponding value of *s*. (**D**) L_2_ error for *I* over intervals of length 6 (*lower*) and 10 (*upper*) as a function of *s*

We can now partially answer one of the questions raised in the Introduction: what are the conditions for adaptation to increase the robustness of slow firing? Figs. 5A, B show that the reduction in the firing rate at steady state is greater when the slope of the *z* nullcline is smaller because the intersections are closer to the threshold. The extreme case of a vertical *z* nullcline corresponds to $s = 0$ in Eq. (3), which implies that the steady-state value of $z = 0$. The adapted system would then be equivalent to (i.e. no better than) the unadapted system. Figure 5B suggests that, in addition to a SNIC, adaptation needs to be sufficiently strong for the *f*–*I* curve to be linear over a large region.

To investigate this quantitatively, we fit straight lines to the steady-state *f*–*I* curves for several values of *s* over intervals of length 6 (Fig. 5C) or 10 and plotted the L_2_ error as a function of *s* for both long and short intervals (Fig. 5D). The error generally decreases with *s*, and larger *s* is needed for linearity over longer intervals. The non-monotonic behavior for small *s* is a consequence of the shapes of the curve of $\langle x \rangle$ and the *s* nullcline (Fig. 5A). When *s* is small, the nullcline intersects the curve of $\langle x \rangle$ on its linear portion, which helps to linearize the adapted *f*–*I* curve. (However, there is little reduction of firing rate, as the behavior of the *f*–*I* curve is dominated by the properties of the fast subsystem.) When *s* is large, the nullcline intersects the vertical portion of $\langle x \rangle$, which again facilitates linearization. For intermediate values of *s*, however, the intersections occur along the nonlinear portion of $\langle x \rangle $, especially for larger *I*, which inhibits linearization. These geometric relationships will play an important role later, when we address systems without a SNIC.

### Approximating the Adapted Firing Rate by Averaging

**Fig. 6:**
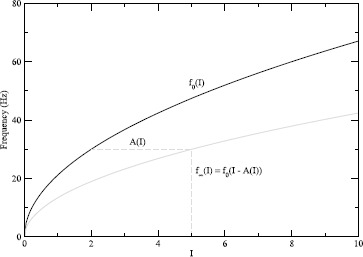
Schematic of stretching. The assumed unadapted firing rate, $f_{0}(I) = 30 \sqrt{0.5 I}$ (*black, solid*) and the adapted firing rate, $f_{\infty}(I) = f_{0}(I - A(I))$, where $A(I) = 0.6 I$ (*gray, dashed*). The *horizontal dashed line* is equal in length to $A(I)$, indicating that $f_{\infty}$ is $f_{0}$ shifted by $A(I)$ ($I = 5$ shifted back to $I = 2$). Thus, $f_{\infty}(I)$ can be viewed as a stretched version of $f_{0}(I)$

Figure 4 also shows that the effect of the increase in *z* is to walk the trajectory back towards the threshold (SNIC). That is, following [8], we write

$$ f_{\infty}(I) \approx f_{0}\bigl(I - A(I)\bigr), $$

where $f_{0}(I)$ is the unadapted firing rate, $f_{\infty}(I)$ is the steady-state adapted firing rate, and $A(I)$ is the adaptive current. (The function $A(I)$ includes implicitly the more direct dependence of the adaptive current on firing rate, *f*.) The walking back is illustrated schematically in Fig. 6. Equivalently, this can be interpreted as saying that the effect of $A(I)$ (which is just *z* in HR) is to stretch the *f*–*I* curve, mapping the interval of low-frequency firing near the SNIC to larger values of *I*. This would both lower and linearize the *f*–*I* curve. In the analysis below, we will relate this geometric picture to the dynamics of the system by a fast-slow analysis of the equations (averaging), with special attention to the role of the SNIC.

The adapted firing rate predicted by the method of averaging can be expressed as

4 $$ f_{\mathrm{pred}}(I) = f_{0}\bigl(I - A(I)\bigr). $$

The approximate firing rate $f_{\mathrm{pred}}$ is calculated for HR as follows: Obtain the curve $\langle x\rangle(z,0)$ (thick red curve in Fig. 4A) by averaging *x* over a spike at *z* for a range of values of *z*. The $\langle x\rangle(z,I)$ curves for other values of *I* (the other thick colored curves in Fig. 4A) are obtained by translating $\langle x\rangle(z,0)$ by *I*. Finally, the intersection of $\langle x \rangle$ with the *z* nullcline is calculated. The value of *z* at that intersection is substituted for $A(I)$ in Eq. (4) to obtain $f_{\mathrm {pred}}(I)$. This should be a good approximation to the firing rate $f_{\infty}(I)$, which is calculated numerically by integrating the full system to steady state, provided averaging is justified, i.e. provided *ε* in Eq. (3) is small.

Equation (4) incorporates the assumption that the firing rate depends only on the applied current as modified by the adaptive current, $A(I)$; in addition we assume that *A* is an increasing function of *I* and that $A(I) = 0$ at the threshold value of *I*, $I_{*}$. The last is reasonable because the adaptation current responds to spike activity, and any baseline current could be absorbed into the other currents. Our goal is to predict the steady-state adapted firing rate but, in contrast to [8, 9], do not address the transient approach to the steady state.

#### Taylor Expansion Method

The approach in [9] was in essence to use a Taylor series to calculate a linear approximation, $f_{\mathrm{taylor}}$, to $f_{\mathrm{pred}}$:

5 $$ f_{\mathrm{taylor}}(I) = f^{\prime}_{\mathrm{pred}}(I_{\star}) (I - I_{\star}). $$

Formally differentiating Eq. (4) gives

6 $$ f^{\prime}_{\mathrm{pred}}(I_{\star}) = f'_{0}\bigl(I_{\star}-A(I_{\star })\bigr) \bigl(1-A'(I_{\star})\bigr). $$

A problem immediately arises: we don’t know *a priori* whether $f^{\prime}_{\mathrm{pred}}(I_{\star})$ exists because it involves $f'_{0}(I_{\star})$, which is infinite because $A(I_{\star}) = 0$ by assumption. We show below that $f^{\prime}_{\mathrm{pred}}(I_{\star})$ is finite. Indeed, the heart of [9] was an argument that this infinite derivative of $f_{0}$ at threshold is not only harmless but is exactly what is needed to linearize $f_{\mathrm{pred}}$. We reproduce the argument here by formally averaging the *z*-equation to estimate $A'(I)$, which for HR is just $\frac{dz}{dI}$. The averaged equation for *z* is

7 $$ \frac{dz}{dt} = \epsilon\bigl(s\bigl(\langle x \rangle- \bar {x}\bigr) -z \bigr), $$

where

8 $$ \langle x \rangle= \langle x \rangle(z,I) = {1 \over T(z, I)} \int_{0}^{T(z, I)} x(t;z, I)\,dt $$

and $T(z, I)$ is the period of the limit cycle for those values of $z, I$.

Since we seek the effect of the adaptive current on the full system at steady state, we set the right hand side of equation (7) to zero,

9 $$ s\bigl(\langle x \rangle(z,I) - \bar{x}\bigr) - z = 0, $$

and indicate explicitly that $\langle x \rangle$ is a function of *z* and *I* along the $\langle x \rangle$–*z* curve in Fig. 4. The solution gives the *z* value of the intersection of the *z*-nullcline with the average curve $\langle x \rangle$.

Next, we implicitly differentiate Eq. (9) with respect to *I* and solve for $\frac{dz}{dI}$, which is needed for the Taylor expansion:

$$s \biggl( \frac{\partial\langle x \rangle}{\partial I} + \frac {\partial \langle x \rangle}{\partial z} \frac{dz}{dI} \biggr) = \frac{dz}{dI} $$

or

10 $$ \frac{dz}{dI} = { s{\partial\langle x \rangle\over \partial I} \over { 1 - s{\partial\langle x \rangle\over \partial z} }}. $$

Since the effects of *I* and *z* are equal in magnitude but opposite in sign we can simplify Eq. (10) using

$${\partial\langle x \rangle\over \partial z } = -{\partial\langle x \rangle\over \partial I }, $$

to get

11 $$ \frac{dz}{dI} = { s{\partial\langle x \rangle\over \partial I} \over { 1 + s{\partial\langle x \rangle\over \partial I} }} \equiv A'(I). $$

We now substitute for $A'(I)$ in Eq. (6):

12 $$ f^{\prime}_{\mathrm{pred}}(I_{\star}) = \lim _{I \rightarrow I_{\star}} f^{\prime}_{0}(I) \biggl( 1- \frac{ s \frac{\partial\langle x \rangle}{\partial I} (0,I)}{ 1 + s\frac{\partial\langle x \rangle}{\partial I}(0,I)} \biggr), $$

again using $A(I_{*}) = 0$. We need the factor in big parentheses to →0 rapidly enough to balance $f'_{0}$, which →∞ as $I \rightarrow I_{*}$. To evaluate this expression, we use the observation in [9] that, if there is a SNIC at $I=I_{\star}$, the time-average of *x* is proportional to the firing rate because the spike shape does not change much as *I* increases from $I_{*}$; only the interspike interval changes (increases). In other words, the integral in Eq. (8) is nearly independent of *I*. These considerations give the approximation

13 $$ \langle x \rangle(z,I) \approx\beta f_{0}(I - z), $$

where *β* is constant. (Note that $\langle x \rangle$ appears to inherit the square-root behavior of $f_{0}$ near the threshold in Fig. 2B.) Thus $\frac{\partial\langle x \rangle }{\partial I } (0,I) \approx\beta f'_{0}(I)$, and

14 $$\begin{aligned} f^{\prime}_{\mathrm{pred}}(I_{\star}) \approx& \lim_{I \rightarrow I_{\star}} f^{\prime}_{0}(I) \biggl( 1- \frac{ s \beta f^{\prime}_{0} (I)}{ 1 + s \beta f^{\prime}_{0} (I)} \biggr) \\ = & \lim_{I \rightarrow I_{\star}} \frac {f^{\prime}_{0}(I)}{1 + s \beta f^{\prime}_{0} (I)} \\ = & \frac{1}{s \beta}, \end{aligned}$$

where the third line uses the assumption that $\lim_{I \rightarrow I_{\star}} f_{0}^{\prime}(I) = \infty$ because of the SNIC.

It is now safe to substitute in Eq. (5) to obtain

15 $$ f_{\mathrm{taylor}}(I) \approx \frac{1}{s \beta} (I-I_{\star}). $$

Thus, $f_{\mathrm{taylor}}$, which is linear by construction, has a slope that decreases with *s*, in agreement with Fig. 5. However, it will only be a good approximation to $f_{\mathrm{pred}}$ when $f_{\mathrm{pred}}$ is in fact nearly linear. This analysis does not tell us when that is true, but Fig. 5D shows that $f(I)$, which should be well approximated by $f_{\mathrm{pred}}(I)$ because *ε* in Eq. (3) is small, may not be very linear unless *s* is large, and thus may not be well approximated by $f_{\mathrm{taylor}}$. The Taylor approximation also relies on $f_{0}^{\prime}(I_{\star}) = \infty$ and so does not account for linearization by adaptation in systems without a SNIC, which will be illustrated below.

#### Mean Value Theorem Method

An alternative to the Taylor expansion of $f_{\mathrm{pred}}$ that avoids the above problems is to apply the mean value theorem to $A(I)$ and show that $f_{\mathrm{pred}}$ is approximately a stretched version of $f_{0}$. Using again our assumption that $A(I_{\star}) = 0$ we have

$$ A(I) = A^{\prime}(\tilde{I}) (I - I_{\star}), $$

where *Ĩ* is in some closed interval $[I_{\star}, I^{\star}]$ and depends on *I*. $A^{\prime}(I)$, given in Eq. (11), satisfies $A^{\prime}(\tilde {I}) \rightarrow1$ as $s \rightarrow\infty$. Moreover, this convergence is uniform if we make the mild assumption that the mean membrane potential decreases monotonically with the adaptation current:

##### Claim 1

*Given any interval* $[I_{\star}, I^{\star}]$ *on which* $\langle x \rangle$ *is a monotonically decreasing function of* *z* (*cf*. *Fig*. 5), $A'(I) \rightarrow1$ *uniformly as* $s \rightarrow\infty$

*Proof*: *As illustrated in Fig*. 5, $-{\partial\langle x \rangle\over \partial z}$ *is bounded below*, *and this can be assumed to hold for any reasonable neural model*. *Then* $\vert-{\partial \langle x \rangle\over \partial z} \vert\geq K$ *for some* $K>0$ *and*

16 $$\begin{aligned} \bigl\vert 1 - A'(I) \bigr\vert =& \biggl\vert 1 - { s{\partial\langle x \rangle\over \partial I} \over { 1 + s{\partial\langle x \rangle\over \partial I} }} \biggr\vert \\ =& \biggl\vert { 1 \over { 1 + s{\partial\langle x \rangle\over \partial I} }} \biggr\vert \\ =& \biggl\vert { 1 \over { 1 + s(-{\partial\langle x \rangle\over \partial z}) }} \biggr\vert \\ \leq& {1 \over sK}. \end{aligned}$$

Since $A^{\prime}(I)$ is bounded by 1, Claim 1 implies that for any $\delta> 0$ there exists an $s_{0} > 0$ such that

$$ (1 - \delta) \leq A^{\prime}(I) \leq1 $$

for all $s > s_{0}$ and all *I* in $[I_{\star}, I^{\star}]$. Together with $A(I_{\star}) = 0$ this implies that $A(I) \rightarrow I - I_{\star}$ uniformly on $[I_{\star}, I^{\star}]$ as $s \rightarrow\infty$. Formally, for any *η* such that $0 < \eta< 1$, there exists an $s_{0} > 0$ such that

$$ (1 - \eta) (I - I_{\star}) \leq A(I) \leq(I - I_{\star}) $$

for all *I* in $[I_{\star}, I^{\star}]$ and $s \geq s_{0}$.

Then, assuming $f_{0}$ is monotonically increasing and rewriting

$$\begin{aligned} f_{\mathrm{pred}}(I) = & f_{0}\bigl(I - A(I)\bigr) \\ = & f_{0}\bigl(I_{\star}+ (I - I_{\star}) - A(I)\bigr) \end{aligned}$$

we have our main result:

17 $$ f_{0}(I_{\star}) \leq f_{\mathrm{pred}}(I) \leq f_{0}\bigl(I_{\star}+ \eta(I - I_{\star})\bigr). $$

**Fig. 7:**
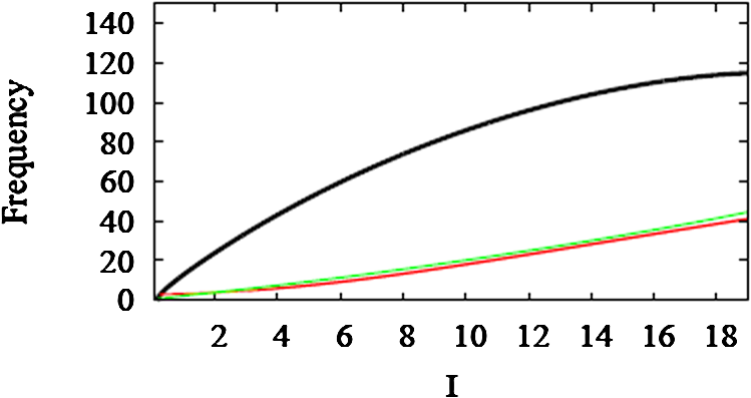
Predicted and actual *f*–*I* curves. The predicted *f*–*I* curve $f_{\mathrm{pred}}(I)$ (*red*) estimated by averaging is a good approximation to the true *f*–*I* curve $f(I)$ (*green*) for the HR model. The *black line* is the unadapted firing rate, $f_{0}$ ($s = 22$)

In words, $f_{\mathrm{pred}}$ is non-negative and bounded by $f_{0}$ evaluated at values of *I* scaled back towards $I_{\star}$, as suggested by Fig. 6. Linearity here comes from stretching the *I* axis, not from assuming that $f_{0}^{\prime}(I_{\star}) = \infty$ as in the Taylor series analysis. Moreover, the region of approximate linearity grows as *s* grows, consistent with Fig. 5D. Figure 7 shows that $f_{\mathrm{pred}}(I)$ is a good approximation to $f(I)$ over a large interval.

The arguments in this section do not rely on the SNIC property, $f^{\prime}_{0}(I_{\star}) = \infty$, but if a SNIC is present in the unadapted system it would contribute to linearization of the adapted *f*–*I* curve. We can see this by using the approximation $\langle x \rangle\propto f$ (Eq. (13)), which is good in the SNIC case, and rewrite $A^{\prime}(I)$ as

18 $$ A^{\prime}(I) = \frac{ s \beta f'_{0} (\tilde{I})}{1 + s \beta f'_{0}(\tilde {I}) }. $$

If $f_{0}^{\prime}(I_{\star}) = \infty$ (or is large because the unadapted system is near one with a SNIC), then $f^{\prime}_{0}(\tilde{I})$ will also generally be large, and *s* will not need to be large to make $A^{\prime}(I)$ near 1.

### Adaptation When the Unadapted System Does Not Have a SNIC

**Fig. 8:**
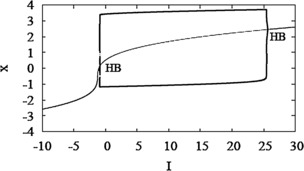
Modified HR system with HB instead of SNIC. Fast subsystem bifurcation diagram with respect to *z*. The change was accomplished by shifting the *x*-nullcline to the right by 0.13 (see *θ* in Table 1)

**Fig. 9:**
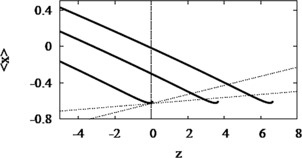
Curves of $\langle x \rangle$ in modified HR with HB. Averaged *x* curves, $\langle x \rangle$, (*thick solid*) correspond to $I = -0.8, 2.8$, and 5.8, increasing from the left. *z*-nullclines (*dotted*) correspond to =20 (*steeper*) and 60. Other parameters as in Fig. 8

**Fig. 10:**
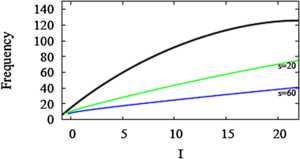
Firing rate curves for modified HR. Adding the adaptive variable *z* to the modified HR system of Fig. 8 makes the *f*–*I* curve shallow and linear. This shows that a SNIC in the unadapted system is not necessary for adaptation and linearization, but larger *s* values are required (compare to Fig. 5B)

Figure 5 shows that having a SNIC in the unadapted HR system (Eqs. (1), (2)) is not sufficient for a linearized firing rate; the adaptation also has to be strong. In this section we show by example, a modified HR model that lacks a SNIC, that a SNIC is not necessary for linearization. The bifurcation diagram of the modified system (Fig. 8) shows that the low-*I* threshold is now a Hopf bifurcation (HB). As a result, the slope $\frac{\partial\langle x \rangle}{\partial z}$ is not infinite even when *s* is large (Fig. 9). Nonetheless, the *f*–*I* curve is linearized and the firing rate is robustly suppressed for large *I* when adaptation is included with sufficiently large *s* (Fig. 10). The adapted frequency is robustly held near the firing rate that the unadapted system exhibits at threshold, which is about 5 Hz, rather than 0. In other examples (not shown) we have found initial firing rates as high as 20 Hz.

### Generalizing to a Conductance-Based Model

In this section, we consider a conductance-based model for adaptive current and apply averaging to approximate the firing rate curve. The model is based on Morris–Lecar [2, 11] with an added adaptive current, $g_{z} z(v-E_{z})$, which has a gating variable *z* that is slower than the other two variables, *v* and *n*:

19 $$\begin{aligned} \frac{dv}{dt} =&-I_{\mathrm{ion}}(v,n)+ I - g_{z} z(v-E_{z}), \\ \frac{dn}{dt} =&g(v,n), \\ \frac{dz}{dt} =& \varepsilon\bigl(h(v) -z\bigr), \end{aligned}$$

where

$$\begin{aligned} I_{\mathrm{ion}} = & - g_{ca} m_{\infty}(v) (v - v_{ca}) - g_{k} n(v - v_{k}) - g_{l}(v - v_{l}), \\ g(v,n) =& \lambda(v) \bigl(n_{\infty}(v)-n\bigr), \\ h(v) =& \frac{1}{1 + e^{- (\frac{v - \bar{v}}{s} )}} , \\ m_{\infty}(v) =& 0.5 \biggl(1+\tanh\biggl(\frac{v-v_{1}}{v_{2}} \biggr) \biggr) , \\ n_{\infty}(v) =& 0.5 \biggl(1+\tanh\biggl(\frac{v-v_{3}}{v_{4}} \biggr) \biggr) , \\ \lambda(v) =& {\phi}\cosh\biggl(\frac{v-v_{3}}{2v_{4}} \biggr) . \end{aligned}$$

Parameter values are in Table 2.

**Table 2:** **Parameter values for SNIC and Hopf bifurcations with Morris–Lecar model**

	SNIC	Hopf
*I*	[40,100]	[58,80]
$g_{z}$	4 nS	6 nS
$v_{k}$	$-84~\mbox{mV}$	$-84~\mbox{mV}$
$v_{l}$	$-60~\mbox{mV}$	$-60~\mbox{mV}$
$v_{ca}$	$120~\mbox{mV}$	$120~\mbox{mV}$
$g_{k}$	$8~\mbox{nS}$	$8~\mbox{nS}$
$g_{l}$	$2~\mbox{nS}$	$2~\mbox{nS}$
$g_{ca}$	$4~\mbox{nS}$	$4~\mbox{nS}$
$c_{m}$	$22~\mu\mbox{F}$	$22~\mu\mbox{F}$
$v_{1}$	$-1.2~\mbox{mV}$	$-1.2~\mbox{mV}$
$v_{2}$	$18~\mbox{mV}$	$18~\mbox{mV}$
$v_{3}$	$12~\mbox{mV}$	$4~\mbox{mV}$
$v_{4}$	$17~\mbox{mV}$	$20~\mbox{mV}$
*s*	2	1.2
*v̄*	−17	18
*ε*	0.0001	0.0001
*ϕ*	0.066667	0.066667

#### Morris–Lecar with SNIC

As noted in [9], the averaged driving force for the adaptive current, $\Delta=\langle v-E_{z} \rangle$, is nearly constant as long as the spike width is small compared to the interspike interval, which will hold if the firing rate is not too high. If we assume that Δ is constant, we can transform the system (19) into a form similar to HR by rescaling the adaptation current using $w = g_{z} z\Delta$:

20 $$\begin{aligned} \frac{dv}{dt} = & -I_{\mathrm{ion}}(v,n)+ I - w, \\ \frac{dn}{dt} = & g(v,n), \\ \frac{dw}{dt} =& \varepsilon\bigl(g_{z} h(v){\Delta} - w\bigr). \end{aligned}$$

**Fig. 11:**
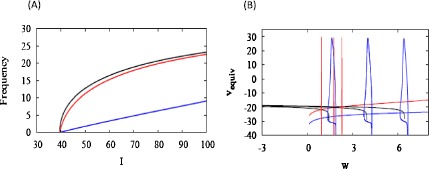
Conductance-based Morris–Lecar model with SNIC and adaptive current. (A) Adaptation and linearization of the *f*–*I* curve are seen for large adaptive conductance ($g _{z} = 4\mbox{ nS}$, *blue*), but not for small conductance ($g_{z} = 0.2\mbox{ nS}$, *red*). (B) Curves of $v_{\mathrm{equiv}}$ are shown for equally spaced values of *I*, $41, 43.5, 46$ pA, increasing from left to right. Each trajectory evolves closely around the intersection point of the equivalent voltage curve and the *w*-nullcline for each *I*. To keep the diagram simple, we have plotted all the *w* nullclines with $\Delta= \Delta(I_{\star}) = -54\mbox{ mV}$

Averaging over a spike period we have

$$\frac{dw}{dt}= \varepsilon\bigl(g_{z} \langle h \rangle (w,I) \Delta-w\bigr), $$

and at steady state

21 $$ w = g_{z} \langle h \rangle\Delta, $$

which is equivalent to Eq. (9) for *z* in HR, except that we now have to average a nonlinear function of *v* rather than *v* itself. As for HR, we implicitly differentiate with respect to *I* to obtain

$$\frac{dw}{dI} = \frac{ g_{z} \Delta\frac{\partial\langle h \rangle}{\partial I}}{{ 1 + g_{z} \Delta\frac{\partial\langle h \rangle }{\partial I} }}, $$

which is equivalent to Eq. (11) with the conductance $g_{z}$ of the adaptation current playing the role of *s* in HR, and the argument used for the HR case goes through. When $g_{z}$ is large, the ML system exhibits linearization and strong adaptation (Fig. 11A). As for HR, the *f*–*I* curve predicted by averaging agrees well with the actual *f*–*I* curve of the full system (not shown).

In order to generalize the geometrical analysis of HR in Fig. 5 to ML (Eq. (20)), in particular to plot the *w* nullcline, we need to define the equivalent voltage, $v_{\mathrm{equiv}}$, as in [12, 13]:

22 $$ h\bigl(v_{\mathrm{equiv}}(w, I)\bigr) = \frac{1}{T(w,I)} \int_{0}^{T(w,I)} h\bigl(v(t)\bigr) \,dt. $$

In addition, we need to evaluate Δ. Although we assumed that Δ was constant to derive Eq. (20), it does vary somewhat with *I*. This would require using different *w* nullclines for each value of *I*. However, we have found that it is sufficiently accurate to choose one value for all *I*, $\Delta(I_{\star})$, in plotting Fig. 11B. Note that the locations of the trajectories are accurately predicted by the intersections of the *w* nullclines with $v_{\mathrm{equiv}}$.

As in the HR case, the $v_{\mathrm{equiv}}$ curve is vertical near the SNIC and for the same reasoning evoked for Eq. (13). Also as in HR, a linear *f*–*I* curve with substantial reduction in firing rate is obtained only when the *w* nullcline intersects the equivalent voltage curve along the vertical portion (blue curves), which only happens when $g_{z}$ is sufficiently large. When this holds, the *w* nullcline picks off equally spaced values of *w* for equally spaced values of *I*. Assuming again that Δ is approximately constant, this implies that the adaptive current is linear in *I*, and this in turn yields the linear *f*–*I* curve. In fact, the adaptive current is linear for the large conductance but is nonlinear for the small conductance (not shown).

#### Morris–Lecar with HB

**Fig. 12:**
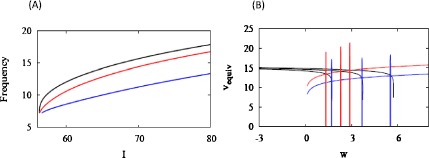
Conductance-based Morris–Lecar model with HB and adaptive current. (A) Adaptation and linearization of the *f*–*I* curve are also seen in this case for large conductance ($g=6\mbox{ nS}$, *blue*), but not small conductance ($g=1\mbox{ nS}$, *red*). (B) Curves of $v_{\mathrm{equiv}}$ are shown for $I=58, 60, 62$ pA, increasing from left to right. Each trajectory evolves closely around the intersection point of the equivalent curve and the *w*-nullcline. All *w* nullclines are plotted with $\Delta= \Delta(I_{\star}) = -63\mbox{ mV}$

As we did for HR, we modify ML so that it has an HB instead of a SNIC. Figure 12A shows that in this case as well, the system exhibits linearization and strong adaptation for $g_{z}$ sufficiently large. Also in this case, linearization results when the *w* nullcline intersects the $v_{\mathrm{equiv}}$ curve on its vertical portion (Fig. 12B). Because Δ can no longer be assumed to be constant in the frozen system, we cannot apply the rescaling used to obtain Eq. (20), and hence cannot use the argument of Claim 1 to predict linearization of *f*–*I*. Also, with Δ non-constant, the linearity of *w* does not imply linearity of the adaptive current. Nonetheless, *f*–*I* and the adaptive current are linear in *I* when $g_{z}$ is large (the latter is not shown).

How can the results be so similar to the SNIC case when none of the assumptions needed to derive the properties of the SNIC case hold? One might think that it is because the system with HB is near one with a SNIC, but the time course of *V* near threshold is very different from the SNIC case—the spikes are distorted sinusoids with no long interspike interval (not shown).

An important clue is that, even though Δ is not constant in the *unadapted* (frozen) system for the HB case, Δ is very close to a constant in the *adapted* system for a large range of *I* near threshold, and that constant is $\Delta(I_{\star})$. This can be seen from Fig. 12A, where the adapted *f*–*I* curve for large conductance samples values of *f* over $I = [58, 80]$ that correspond to values of *f* achieved over $I = [58, 63]$ in the unadapted system. The values of Δ are correspondingly limited to those attained in the unadapted system in the narrower range of *I*. In view of this, the near linearity of *w* does imply near linearity of the adaptive current. The near constancy of Δ is further demonstrated by the accurate prediction of the *w* locations of the trajectories in Fig. 12B, in which the *w* nullclines are plotted using $\Delta(I_{\star})$.

The near constancy of Δ in the adapted system could have been predicted *a priori* because, independent of Claim 1, we should expect stretching of the *f*–*I* curve when $g_{z}$ is large from the diagram in Fig. 6. We do not know whether the stretching is linear, but this observation justifies replacing $(v - E_{z})$ in Eq. (19) with $\Delta(I_{\star})$ for the purpose of predicting the behavior of the full, adapted system. The argument of Claim 1, which does not depend on a SNIC or low frequency near threshold, then goes through, giving linear adaptive current and linear *f*–*I* for large $g_{z}$.

A final point that requires explanation is why the $v_{\mathrm{equiv}}$ curve has a nearly vertical portion near the threshold in the HB case. In the SNIC case, this follows from averaging (Eq. (13)) and, biophysically, from the lengthening of the interspike interval, which decreases $\langle V \rangle$, as $I \rightarrow I_{\star}$. This does not occur in the HB case, rather the sigmoidal shape we assumed for *h* comes into play. Mean *v*, and hence mean *h* (RHS of Eq. (22)), will tend to decrease as the SNIC is approached, but this increase may be gradual. However, if $h(v) \approx0$ for *v* at the threshold, which is plausible, $v_{\mathrm{equiv}}$ is forced to drop sharply to the flat region of *h*.

Note that in HR with HB (Fig. 9), where the activation of *z* is linear, $x_{\mathrm{equiv}} = \langle x \rangle$, and the drop in $x_{\mathrm{equiv}}$ is gradual. We have checked that if *h* is made linear in the ML system (Eq. (19)), $v_{\mathrm{equiv}}$ is similarly gradual (not shown). The example of Fig. 9 shows that a vertical drop in $v_{\mathrm{equiv}}$ is not necessary for a linear *f*–*I* curve. We will not attempt to account for all possible cases but conclude merely that a vertical drop in $v_{\mathrm{equiv}}$ is not improbable in the HB case, and, if there is a vertical drop, *f*–*I* will be linearized and stretched when $g_{z}$ is large enough.

## Discussion

### Context

Spike-frequency adaptation in neurons is well-studied in part because it is a basic and ubiquitous feature of neural behavior and in part because it contributes to information processing by networks of neurons. For example, in [6] it was shown to participate in forward masking, and in [14] local fatigue, which includes adaptation, was found to be responsible for switching between percepts in binocular rivalry. This in turn has generated interest in simplified models to facilitate simulation of large networks [8].

Others have focused on the ability of adaptation to linearize the *f*–*I* curve, because adapted neurons show this behavior and also because it has been found to have favorable properties in artificial neural networks for learning [15]. It was argued in [9] that linearization does not need to be imported into the system by assuming that the adaptation current was linear, as in [6]. Rather, linearization is a natural consequence of the square-root behavior of the unadapted *f*–*I* curve, which in turn comes from the presence of a SNIC in the unadapted spiking system.

As in previous analyses, we assume that adaptation is slow so that averaging can be applied, but we ask a different question: how does adaptation make low-frequency firing robust, that is, how is it maintained for a large range of input current? The main result that flowed from this question was that robustness and linearization both arise from adaptation because it stretches out the *f*–*I* curve. In retrospect this is natural because, as we learn in the first week of calculus, linearization is fundamentally a matter of stretching the scale of the independent variable. The role of stretching was previously illustrated in [8], their Fig. 8A, but was not made central to the theory.

Another way to linearize *f*–*I* curves that does not involve adaptation is noise, which can trigger firing at sub-threshold levels of *I* and smooth out a sharp threshold. See for example Fig. 1 in [16]. This is different in effect as well as mechanism from adaptation in that it achieves linearization by increasing firing at low *I* rather than reducing firing rate, so we will not address it further here.

### Comparison to Previous Analyses

We confirmed the results in [9] that a SNIC in the unadapted system fosters linearization and robust reduction in firing rate. However, we showed numerically (Fig. 5D) that, whereas any degree of adaptation will result in linearization of the *f*–*I* curve, the size of the linear region depends continuously on the strength of adaptation (Fig. 5D). This showed that our concern about transferring parameter sensitivity to the adaptation equation was not unfounded and that it has a natural geometric interpretation. If the conductance is too low, then the nullcline of the adaptation variable in the Hindmarsh–Rose (HR) model will be nearly vertical (Fig. 5A), and the adapted system will be little different from the unadapted one (Fig. 5B). Similar but more complex graphs were made for the conductance-based Morris–Lecar (ML) model, in which the adaptation variable nullcline is nonlinear (Figs. 11B, 12B).

For the simple case of HR, in which the adaptation current has no fast voltage dependence (for example, no driving force), we showed further that a SNIC is not necessary if the adaptation conductance is large. If a SNIC is present, however, it would combine with the conductance to mediate linearization, so that the conductance need not be as large (Eq. (18)). The role of adaptation strength is intuitively obvious, and previously published numerical examples of linearization must have tacitly assumed it, but this feature was not revealed in previous analyses.

For ML with a SNIC, where voltage dependence comes into the adaptation current through the driving force ($\Delta= v - E_{z}$ in Eq. (19)), we needed to assume that Δ is nearly constant. As argued in [9], this is likely to be a good approximation when there is a SNIC because *v* is nearly constant during the interspike interval, which dominates the oscillation period. If the unadapted system lacks a SNIC but is sufficiently near one that does have a SNIC, the firing rate would be low, and Δ should again be nearly constant. In [8] a more detailed analysis was carried out of this assumption and ways in which it may fail to hold, but we limit our consideration to cases where this is not a problem in order to focus on the essential features.

In addition to a SNIC, the analysis in [9] used the approximation that the adaptation current is proportional to the firing rate, as did [6] and [8]. This approximation was argued in [9] to follow from averaging the equation for the adaptation variable. In [8] it was shown that this may not always hold, depending on the voltage or calcium dependence of the adaptation current, and it was stated as a separate assumption (their Eq. (5.5)). This assumption is equivalent to the approximation we used in recasting the argument from [9], $\langle x \rangle(z,I) = \beta f_{0}(I - z)$ (Eq. (13)), because *z* is proportional to $\langle x \rangle$. (See Eq. (9); the offset *x̄* is inconsequential as it could be absorbed into the applied current *I* and shifted to the *x* equation.) Note that we did not use this assumption in deriving the role of the adaptation conductance (Claim 1 and following text), but only the milder assumption that $\langle x \rangle$ decreases monotonically with *z*.

The analysis of [9] essentially employed a linear approximation obtained by Taylor expansion around the SNIC. However, the Taylor approximation is only good when the adapted firing rate is nearly linear, and this is only assured when adaptation is strong. In [8], it was assumed tacitly that the slope of the unadapted *f*–*I* curve is sufficiently large in a sufficiently large neighborhood of threshold, as expected for a SNIC.

We circumvented this difficulty by applying the mean value theorem to the adaptation current, rather than approximating the firing rate itself. We showed that the adaptation current is nearly proportional to the applied current when the adaptation conductance is large (Claim 1). Our argument provided a uniform bound on the deviation from linearity as adaptation strength increases and also made the role of stretching more apparent (Eq. (17)). We did not have to make any assumption about the frequency dependence of the adaptation current.

Our analysis of stretching applies to HR in the type 2 (HB) case, but the strength of adaptation will generally have to be larger to achieve a linear *f*–*I* curve because there is no help from a SNIC (Fig. 10). Also, the firing rate defended by adaptation will not be 0, but whatever the threshold firing rate of the unadapted system happens to be.

However, our method does not apply to conductance-based, type 2 neurons because the voltage dependence of the adaptation current (present at least in the driving force) prevents use of the scaling argument we needed to transform the ML system to HR form. Nonetheless, adaptation and linearization can occur for sufficiently large adaptation conductance, as illustrated in Fig. 12. This happens because Δ is nearly constant for the *adapted* system even though it varies in the *unadapted* system. That in turn follows from the stretching, possibly nonlinear, of the *f*–*I* curve by the adaptive current (Fig. 6). Finally, this allows us to replace the type 2 system by one with constant driving force, and Claim 1 gives linear stretching as for type 1. The approximation breaks down for large enough *I*, but in practice it is good for a large range.

Our formulation for conductance-based models, with an adaptation current that is linear in a single gating variable, may not cover all possible cases, but it does include many typical ones, including two cases considered in [8] and [9], a voltage-dependent M-type $\mathrm{K}^{+}$ current and an AHP current with a conductance that is linear in calcium. For adaptation currents with more complex voltage dependence, such as a slowly inactivating $\mathrm {Na}^{+}$ current with fast gating variables, our theory may not apply even in the presence of a SNIC because the scaling argument used to derive Eq. (20) may not be valid even approximately.

### Heuristic Summary

Consider an adaptation current of the form

$$ A = g_{z} z (v - E_{z}), $$

where *z* is slow and has a monotonic activation function, *h* (typically a sigmoid):

$$ \dot{z} = \varepsilon\bigl(h(v) - z\bigr). $$

Let the unadapted firing rate be $f_{0}$. The steady-state adapted firing rate $f_{\infty}$ is approximated by

$$ f_{\infty}(I) \approx f_{0}\bigl(I - A(I)\bigr). $$

WLOG let $f_{0}(0) = 0$ and $A(0) = 0$. Then, if $g_{z}$ is sufficiently large, $A(I) \approx a I$, where $a \lessapprox1$, and

$$ f_{\infty}(I) \approx f_{0}\bigl(I (1 - a)\bigr). $$

The larger $g_{z}$ is, the closer *a* will be to 1, and the more strongly will the *I* axis be mapped toward 0, resulting in a linearized adaptation curve.

This result depends on $v- E_{z}$ being nearly constant, which will hold if the unadapted system has a SNIC; together with large $g_{z}$ this constitutes a sufficient set of conditions. If the unadapted system does not have a SNIC but has low-frequency firing near threshold, or an interspike interval that is much larger than the spike width, then large $g_{z}$ is sufficient. Even if none of the above conditions apply, large $g_{z}$ may be sufficient in many cases, as illustrated in Fig. 12. As observed in [8], there is unlikely to be a general theory to cover all type 2 systems.

### Extensions

The SNIC plus slow negative feedback scenario is general, and should apply to many situations other than neuronal adaptation. One well-known candidate system is ER-driven calcium oscillations, which may exhibit frequency encoding of stimulus strength (ligand concentration) [17]. This can be achieved if the oscillation threshold is generated by a SNIC but is not robust. It has been suggested that control of oscillation frequency can be made more robust by adding a slow process to inhibit the IP3 receptor, specifically a calmodulin-dependent phosphorylation in Fig. 3B, [18]. Since calmodulin is activated by calcium, it would qualify as an activity-dependent adaptation process analogous to neuronal adaption, but this has not to our knowledge been modeled in detail.

Other means of achieving robustness have been considered theoretically. One is to average cell properties over a large population [19, 20]. That works well for a uniform but sloppy population of cells that need to synchronize to carry out a stereotypical task. For neuronal networks, in which individual cells may need to be constrained, the mechanism studied here, making parameters into variables, is more appropriate. A previous line of investigation had already introduced dynamic control of parameters, but differed in locating control at the level of gene expression [21]. Such regulation is slow, requiring tens of minutes to hours, whereas adaptation operates on the sub-second time scale.

What if a given adaptation process is not sufficiently strong? One solution is to increase the strength, but, if this is not feasible, an alternative is to make a parameter of the adaptation process itself into another slow variable. Chaining multiple negative feedback loops together should lead to a multiplicative improvement. This is also appropriate from the point of view of evolution, which cannot afford to rip out the knitting and start over. It is better to keep moving forward by adding new layers of control.
